# Membrane Structure Obtained in an Experimental Evolution Process

**DOI:** 10.3390/life12020145

**Published:** 2022-01-20

**Authors:** María J. Dávila, Christian Mayer

**Affiliations:** Institute of Physical Chemistry, CENIDE, University of Duisburg-Essen, 45141 Essen, Germany; christian.mayer@uni-due.de

**Keywords:** origin of life, bilayer structure, molecular dynamics, aggregation process, selection, evolution

## Abstract

Recently, an evolution experiment was carried out in a cyclic process, which involved periodic vesicle formation in combination with peptide and vesicle selection. As an outcome, an octapeptide (KSPFPFAA) was identified which rapidly integrated into the vesicle membrane and, according to its significant accumulation, is obviously connected to a particular advantage of the corresponding functionalized vesicle. Here we report a molecular dynamics study of the structural details of the functionalized vesicle membrane, which was a product of this evolution process and is connected to several survival mechanisms. In order to elucidate the particular advantage of this structure, we performed all-atom molecular dynamics simulations to examine structural changes and interactions of the peptide (KSPFPFAA) with the given octadecanoic acid/octadecylamine (1:1) bilayer under acidic conditions. The calculations clearly demonstrate the specific interactions between the peptide and the membrane and reveal the mechanisms leading to the improved vesicle survival.

## 1. Introduction

In early prebiotic evolution, membranous boundaries were probably formed by simple amphiphilic molecules, easier to synthesize than phospholipids and able to integrate and accumulate other amphiphilic molecules. They would give rise to aggregation and membrane disruption, as well as to the formation of primitive membrane pores, thus enhancing selective molecular exchange between the first cell-like compartments and their environment [1,2]. The most commonly accepted model of a prebiotic membrane is the one formed by fatty acids, mainly because of their high permeability and their ability to grow by the integration of new fatty acids [3,4,5]. However, fatty acid membranes may also contain other compounds present in prebiotic conditions, improving their stability and permeability [6,7,8,9,10]. It has been shown that vesicles composed of fatty acids together with other single-chain amphiphilic compounds, such as fatty alcohols, are formed over a wider pH range than vesicles composed of fatty acids only, which are stable at a pH close to their pKa [11]. The addition of other prebiotically plausible molecules to fatty acid/alcohol mixtures, such as isoprenoids, improves vesicle stability, even under extreme conditions of high temperatures, pH, and salinity, and even in the presence of divalent cations [12]. Maurer et al. also described an increase in the stability of fatty acid vesicles by adding their glycerol monoacyl amphiphile derivatives [13]. It was also observed that by incorporating oxidized polycyclic aromatic hydrocarbons into the membrane of fatty acid vesicles, an increase in their stability is produced. Moreover, this leads to a decrease in the membrane permeability, thus showing an effect similar to that of cholesterol or other sterols in current membranes [14]. Namani and Deamer reported a specific type of vesicles formed by fatty acids and their corresponding fatty amines, characterized by being stable in strongly basic and acidic pH ranges [15].

One of the most widespread hypotheses of the origin of life, in which the formation of membranous compartments plays a decisive role, is the hot spring hypothesis [16]. It is based on the assumption that lipid-encapsulated polymers with initially random sequences can be synthesized in wet-dry cycling processes. In these processes, protocells subject polymers to combinatorial selection, leading to the emergence of structural and catalytic functions [16,17].

A new hypothesis proposed by Schreiber et al. [18] proposes the early steps of the origin of life in deep-reaching tectonic faults. In this model, the formation of vesicular structures in these water- and CO_2_-filled zones has been proposed. The mechanism of formation includes cyclic phase transition processes between supercritical CO_2_ and subcritical gaseous CO_2_ by periodic pressure variations at a suitable depth [19]. In water/carbon dioxide interface zones, a pool of oligopeptides of variable length is generated by condensation reactions from amino acids in a hydrothermal environment [20]. Those oligopeptides, which have an amphiphilicity similar to the membrane of vesicular structures, will integrate and accumulate, increasing in concentration over time [21]. Based on the above model, Mayer et al. performed experiments with cyclic pressure variations at the water/CO_2_ interface, allowing vesicular structures to form from a mixture of long amine chains and long fatty acids previously proposed by Namani and Deamer [15]. Since the experiments were performed at 120 °C under acidic conditions, the octadecylamine/octadecanoic acid mixture was selected to improve the stability of the resulting vesicles. The continuous peptide accumulation combined with a selection of the vesicles for thermal stability led to an evolution-like process, finally resulting in functional peptides with the specific capability to make vesicles survive. One of the peptides that was particularly successful under these conditions turned out to be an octapeptide with the sequence H-Lys-Ser-Pro-Phe-Pro-Phe-Ala-Ala-OH (KSPFPFAA). In separate experiments, it could be shown that this peptide led to a reduced vesicle size, an increased membrane permeability, and to a significant increase in the thermal stability of the vesicles [22].

The main goal of this study was to understand the particular mechanism of stabilization which has evolved in the pressure-cycling process. More explicitly, we wanted to elucidate the structural changes and interactions that are induced by the presence of the given octapeptide in the vesicle membrane. To this end, we used all-atom molecular dynamics to study the aggregation ability of the peptide KSPFPFAA in the octadecanoic acid/octadecylamine (1:1) bilayer membrane under conditions that mimic an acidic environment.

## 2. Computational Methods

### 2.1. Simulation Systems

#### 2.1.1. Peptide in Explicit Solvent

The conformation of the octapeptide KSPFPFAA was initially fully extended (backbone dihedral angles are 180° except for dihedral angle C–N–C*α*–C of proline, which is determined by its ring structure) and generated by GaussView [23]. To mimic the experimental acidic conditions described above, the peptide carries a net charge of +1 and both termini are charged.

A biphasic octane/water solvent was used to reproduce the behaviour of the octadecylamine/octadecanoic acid bilayer. From the initial fully extended conformation, the peptide was inserted parallel to the *z*-axis in a hydrated octane slab, with the system’s initial dimensions of 6.4 nm × 6.4 nm × 8.6 nm. The resulting system contained 423 octane molecules and 5120 water molecules (chloride and sodium counterions were added to neutralize the charge). For comparative purposes, the peptide molecule was also placed in the centre of an initial cubic box (4.0 nm × 4.0 nm × 4.0 nm) containing 2043 water molecules (again, chloride and sodium counterions were added to neutralize the charge).

#### 2.1.2. Peptides in Octadecylamine/Octadecanoic Acid Bilayer

The peptide aggregation study was carried out with sixteen peptides located perpendicular to the membrane surface in both parallel and antiparallel arrangement at a distance of 2.8 nm. Thus, the peptides translocate in the xy plane forming a 4 × 4 network, ensuring that there is no prior interaction between adjacent peptides. The initial dimensions of the simulation box were 11.4 × 11.4 × 8.6 nm^3^, containing 366 amphiphilic pairs, octadecylamine and octadecanoic acid, and 15,680 water molecules. Both membrane–peptide systems were neutralized with sodium and chloride counterions. The most representative peptide conformation, which was obtained in a clustering process after the peptide simulation in the previously described octane–water system, was used as the initial peptide conformation in each simulation system.

### 2.2. Simulation Setup

All-atom MD simulations were performed using GROMACS package [24], where CHARMM36 force field [25] and TIP3P [26,27] water models were used. Periodic boundary conditions were applied in all directions. The initial systems were minimized to ensure the elimination of any close contacts by using the steepest descent algorithm and equilibrated for 500 ps in the *NVT* ensemble. Afterwards, an equilibration process was carried out for 20 ns and 40 ns in the *NPT* ensemble, for the biphasic octane/water system and for octadecylamine–octadecanoic acid bilayer, respectively. The Berendsen thermostat method [28] was applied for temperature coupling to a temperature bath of 353 K. In the *NPT* ensemble, the Berendsen weak-coupling algorithm [28] was used to maintain the system to a pressure bath of 1 bar. Production dynamics were performed in the *NPT* ensemble at a temperature of 353 K, using the velocity rescaling thermostat [29] with a coupling time of 0.1 ps, and pressure of 1 bar, using the Parrinello-Rahman barostat [30] with a coupling time of 2 ps. All H-bonds were constrained using the LINCS algorithm [31]. The particle mesh Ewald method (PME) was used for computation of the electrostatic forces with a real space cut-off of 1.2 nm [32]. Van der Waals interactions were cut off at 1.2 nm with a switch function starting at 1.0 nm. GROMACS package was also used to analyse the trajectories. All system configurations were visualized using VMD software [33].

## 3. Results

### 3.1. Peptide Conformation into Biphasic Membrane Mimetic

In order to obtain the most representative conformation of the octapeptide KSPFPFAA and to start the simulations in each of the systems described in the previous section, we carried out a 2.5 μs simulation at 353 K, above the membrane transition temperature, with the peptide initially in the extended–coil conformation placed in a hydrated octane slab. For comparative purposes, a simulation was performed under the same conditions as described above, with the peptide in explicit water.

**Figure 1:**
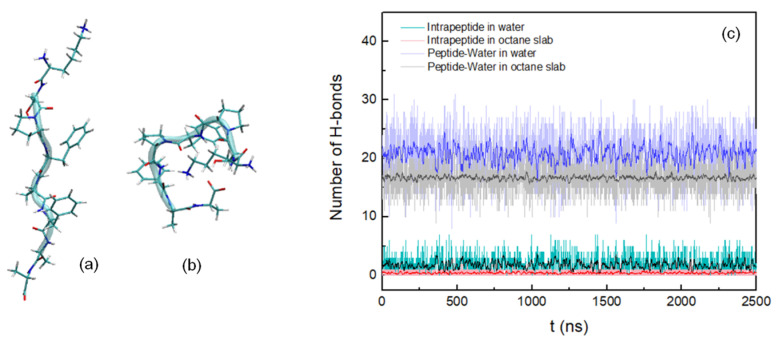
Most representative peptide conformations in the hydrated octane slab (**a**) and in water (**b**). (**c**) Number of hydrogen bonds as a function of time during 2.5 μs: intrapeptide in octane slab, light red; intrapeptide in water, cyan; peptide–water in octane slab, light grey; peptide–water in water, light blue. A running average (window of length 10 ns) was applied to smooth the time series (intrapeptide in octane slab, red; intrapeptide in water, black; peptide–water in octane slab, dark grey; peptide–water in water, dark blue).

After performing a clustering analysis for the octapeptide into the hydrated octane slab with a cut-off of 0.13 nm, the most representative conformation was extracted, being the initial conformation for each of the simulations we performed in this work (Figure 1a). The most representative conformation of the peptide in water with a cut-off of 0.19 nm is shown in Figure 1b.

In the following, the amino acids of the chain will be referred to by a combination of the letter code for the amino acid combined with its position in the peptide chain, so K1 means the first amino acid, A8 the last one. For both systems, the number of intramolecular and peptide–water hydrogen bonds was calculated during the entire simulation, considering their formation with cut-offs of 0.35 nm for the donor-acceptor distance and 30° for the hydrogen-donor-acceptor angle. Thus it was observed that for the peptide insert into the hydrated octane slab, the formation of H-bonds is less frequent than for the peptide in water (Figure 1c). Most of the hydrogen bonds are being formed between the hydroxyl hydrogen of S2 and the carbonyl oxygen of P3 in the first system, while in the second system is dominated by the H-bonds K1-A8, S2-A8, and S2-F4. The results of the formation of hydrogen bonds between peptide and water monitored over the course of the simulations primarily show H-bonds between the amine hydrogen of K1 and the oxygen of water and between the hydrogen of water and the carbonyl oxygen of A8, being predominant in the biphasic membrane mimetic (Table 1).

**Figure 2:**
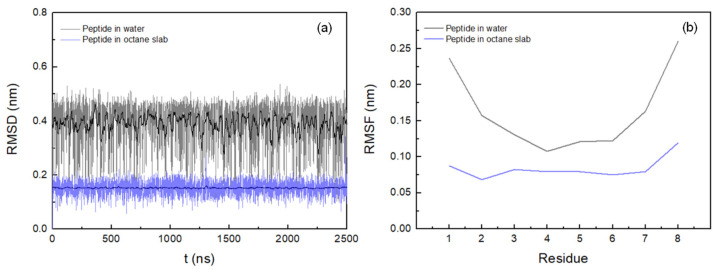
Backbone RMSD (**a**) and RMSF (**b**): peptide in octane slab, blue; peptide in water, grey. A running average (window of length 10 ns) was applied to smooth the time series (peptide in octane slab, dark blue; peptide in water, black).

The initially fully extended structure was used as a reference to calculate the root mean square deviation (RMSD) of the backbone atoms over the course of the trajectory, so the lower RMSD values correspond to the most extended structures. As shown in Figure 2a, the RMSD levels of around 0.15 nm for the peptide in the hydrated octane slab suggest that within 2.5 μs of simulation, with the force field chosen to model the peptide, it did not reach a folded structure. The maximum conformational changes can be found in the peptide in water, increasing the RMSD values up to 0.5 nm. However, strong fluctuations are observed, suggesting a high flexibility and instability of the peptide in this system over the entire MD simulation.

In order to investigate the structural flexibility of the peptide in the octane slab and in water, the backbone root mean square fluctuation (RMSF) was calculated (Figure 2b). The values amount to 0.12 nm in the first system and to 0.26 nm in the second, with the highest RMSF values corresponding to the N- and C-terminal amino acid residues. In the biphasic membrane mimetic, the low RMSF values suggest conformational stability of the peptide in octane slab, showing low flexibility. However, RMSF values of the peptide in water are higher, varying between 0.11 nm and 0.16 nm from S2 to A7, and increasing its values for amino acids directly connected to the terminal residues. Thus, it may indicate that the high flexibility of the terminal residues may also affect the mobility of the adjacent amino acids.

### 3.2. Cluster Process and Pore Formation

**Figure 3:**
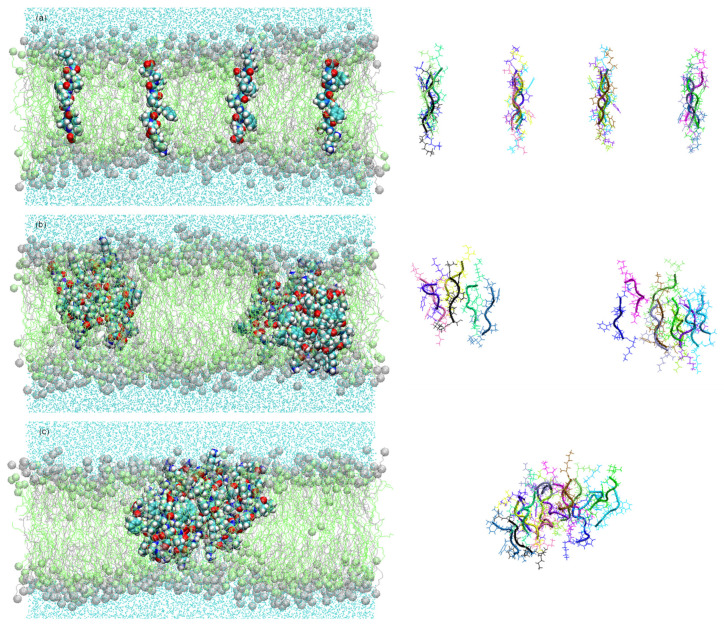
Snapshots of the simulation systems in the peptide aggregation process (peptides are shown as lines with cartoon representations on the right) in antiparallel arrangement at (**a**) 0 ns, (**b**) 100 ns, and (**c**) 450 ns.

In order to obtain information about aggregation and pore formation of the KSPFPFAA peptide, we studied the peptide–peptide and peptide–membrane interactions. This was done in a parallel arrangement of sixteen peptides located perpendicular to the membrane surface, and in an antiparallel arrangement with alternating orientation of the peptides (Figure 3). The method described by Thøgersen et al. [34] was applied where the peptides are positioned at a starting distance of 2.8 nm, thus ensuring no prior interaction between adjacent peptides.

It was observed that in the case of peptides located in antiparallel orientations, they remained embedded in the membrane during 450 ns simulation. Here, peptides are not exchanged between clusters over the entire MD simulation. In general, the membrane embedded peptides show a certain stability of its conformation, thus remaining extended structures (Figure 3). Peptides positioned in parallel arrangement, on the other hand, tend to emerge to the membrane surface, so that after only 100 ns there are no peptides in the transmembrane position. In this arrangement, the peptides rapidly emerge to the surface of the membrane while changing their conformation. Hence, in the transition from embedded peptides to surface-emerged peptides, probably due to their higher structural flexibility, a progressive increase of instability in their conformation is observed. (The results of the parallel configuration are not shown in this work).

**Figure 4:**
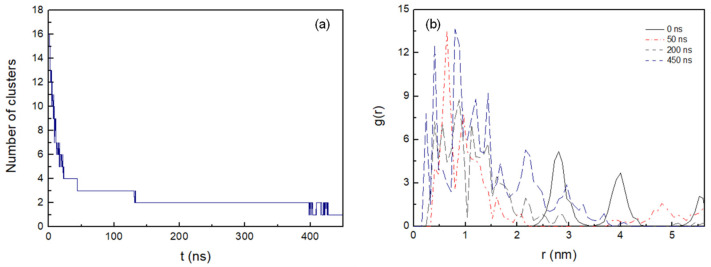
(**a**) Number of the clusters as a function of time formed during the 450 ns MD simulation with cut-off 0.6 nm. (**b**) Intermolecular radial distribution function, g(r), between the mass centres of the peptide pairings at 0 ns, 50 ns, 200 ns, and 450 ns (0.08 nm bin width).

Figure 4a shows the number of clusters formed in the antiparallel configuration during 450 ns MD simulation. The lateral diffusion of the octapeptide allows for rapid dimer formation in the first 8 ns and the stable cluster formation of up to five peptides at just 22 ns. Cluster size increases progressively and completes its total aggregation process at 428 ns.

The radial distribution function, g(r), was used to determine the peptide–peptide interactions during the cluster formation process. It was calculated in the xy plane at 0 ns, 50 ns, 200 ns, and 450 ns for the peptide pairs inserted in the membrane (Figure 4b). A high degree of clustering is observed as the aggregation process evolves, in relation to the initial configuration, with the maximum peaks positioned at 2.8 nm and 4 nm, corresponding to the distances between the mass centres of the peptides initially fixed. At 50 ns, a maximum peak in the radial distribution function appears at 0.65 nm, although, as indicated by its profile, the aggregation process is still incomplete. At 200 ns, the most likely distance between aggregated peptides is 0.93 nm, with several pronounced distribution peaks at 0.77 nm, 0.57 nm, and 0.41 nm. At the end of the aggregation process at 450 ns, several peaks are observed, with the most intense distribution peaks at 0.82 and 0.41 nm, as well as a narrow peak at 0.25 nm. Hence, the radial distribution function profiles obtained during the aggregation process suggest that there is a strong tendency for peptides to pack and cluster in the membrane octadecylamine/octadecanoic acid.

**Figure 5:**
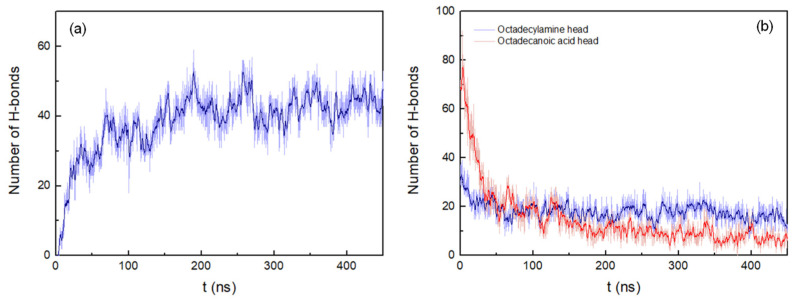
(**a**) Number of hydrogen bonds between peptides pairings. (**b**) Number of hydrogen bonds between octadecylamine and octadecanoic acid head groups and the peptides. Both representations show the results of the system with peptides of antiparallel arrangement up to 450 ns. A running average (window of length 2 ns) was applied to smooth the time series: (**a**), dark blue; (**b**), dark blue (octadecylamine head group–peptide interactions) and red (octadecanoic acid head group–peptide interactions).

The H-bonds between the peptide pairings were also determined, considering a donor-acceptor distance within 0.35 nm and 30° for the hydrogen-donor-acceptor angle (Figure 5a). The number of hydrogen bonds continuously increases during the simulation until stabilizing at around 175 ns. Here it is worth noting the formation of hydrogen bond interactions between the residues K1 and A8 of the peptide pairings, which are present with an occurrence of 36% during the whole MD trajectory. More specifically, these hydrogen bonds are mainly established between the amine hydrogen of K1 and the carbonyl oxygen of A8, which contribute to a greater stabilization of the peptides inserted in the membrane.

The interactions between octadecylamine and octadecanoic acid hydrophilic head groups with the peptides were calculated to obtain information on how these interactions are affected by the peptide aggregation process. The number of hydrogen bonds between the fatty acid head groups and the peptides in the antiparallel arrangement system decreases rapidly up to 54 ns. After that, the interactions deteriorate more slowly until approximately 350 ns. At this point in time, the number of hydrogen bonds begins to stabilize, corresponding to the end of the aggregation process (Figure 5b). However, the decrease in the number of hydrogen bonds is less pronounced for the interactions between the octadecylamine head group and the peptide, showing a slight decrease in the number of H-bonds, decreasing up to 61 nm from the beginning of the aggregation process and stabilizing practically from there until the end of the simulation. Thus, the results seem to indicate that the amine head group forms hydrogen bonds with the peptides less frequently than the fatty acid head group.

**Figure 6:**
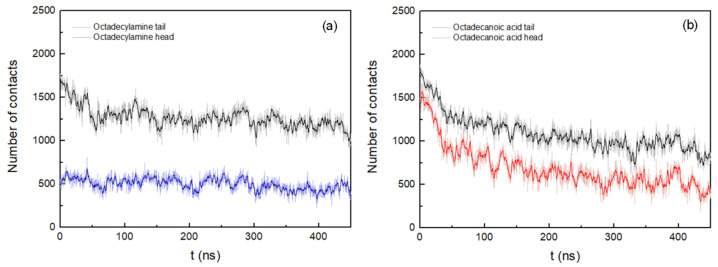
(**a**) Number of atomic contacts between octadecylamine molecules and peptides. (**b**) Number of atomic contacts between octadecanoic acid molecules and peptides. Both representations show the results of the system with peptides in antiparallel arrangement up to 450 ns. A running average (window of length 2 ns) was applied to smooth the time series: (**a**) dark blue (octadecylamine head group) and black (octadecylamine tail region); (**b**) red (octadecanoic acid head group) and black (octadecanoic acid tail region).

The interactions between the amphiphilic membrane molecules and the peptides were also evaluated by calculating the number of atomic contacts for both systems (cutoff 0.6 nm). Figure 6 shows the number of atomic interactions of the hydrophilic head groups (–NH_3_^+^ and –COOH) and hydrophobic tails of octadecylamine and octadecanoic acid with the peptides.

Figure 6a shows that the number of interacting atoms between octadecylamine tail regions and peptides decreases sharply up to 55 ns. After that, only a slight decrease is observed until the end of the simulation. However, the number of contacts between atoms of the octadecylamine head group and the peptides is practically constant during the whole simulation. Obviously, it is not being influenced by the aggregation process. In the interactions between the atoms of the octadecanoic acid and the peptides (Figure 6b), a progressive decrease is observed in most of the 450 ns of the MD simulation; F4 and F6 residues interact the most frequently with the atoms of the octadecanoic acid tail region (Table 2 shows the relative amounts of contacts of the various amino acid positions with the head and tail ends of the octadecylamine and the octadecanoic acid). The number of atomic interactions between the octapeptides and acid tail region also increase, being more pronounced at the beginning of the simulation, with strong fluctuations.

**Figure 7:**
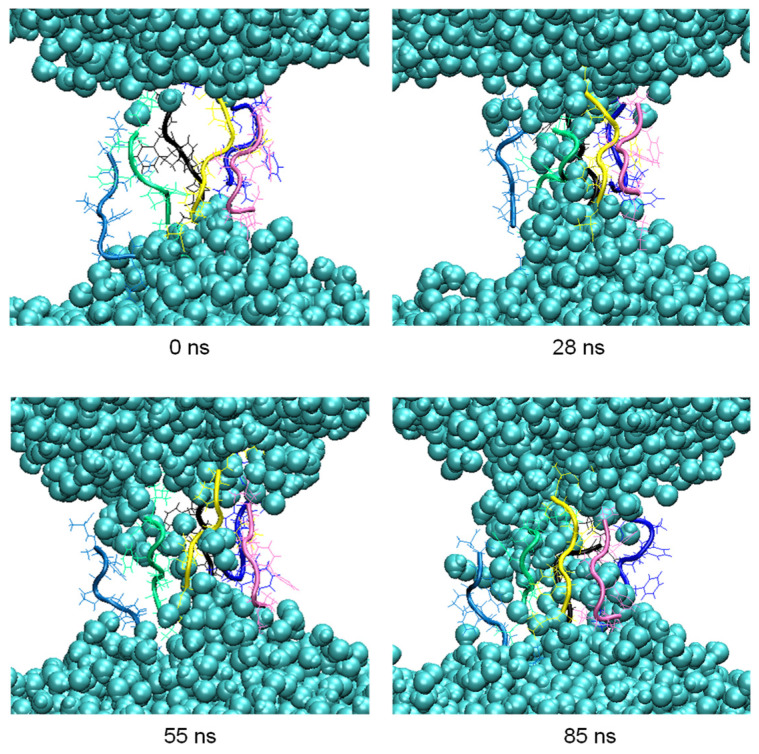
Snapshots of the penetration of the water molecules (blue spheres) into the membrane at relevant times of the 100 ns MD simulation.

During the aggregation process, penetration of water molecules into the membrane was observed. Thus, in the pore formed by six peptides at 200 ns in the clustering process, MD simulations were carried out for another 100 ns to observe this effect more in detail (Figure 7).

The number of hydrogen bonds between the residues of the peptides forming the pore and the water molecules were analyzed in the 100 ns of simulation. The highest occurrences were observed in the terminal residues, K1 and A8, with 30% and 28%, respectively, and S2 with 18%. The difference in the occurrence of hydrogen bond formation between the residues of the octapeptide and the water molecules, and hence the ability of the pore to allow water molecules to flow inside the membrane, is influenced by the different orientations adopted by the side chains of the residues.

**Figure 8:**
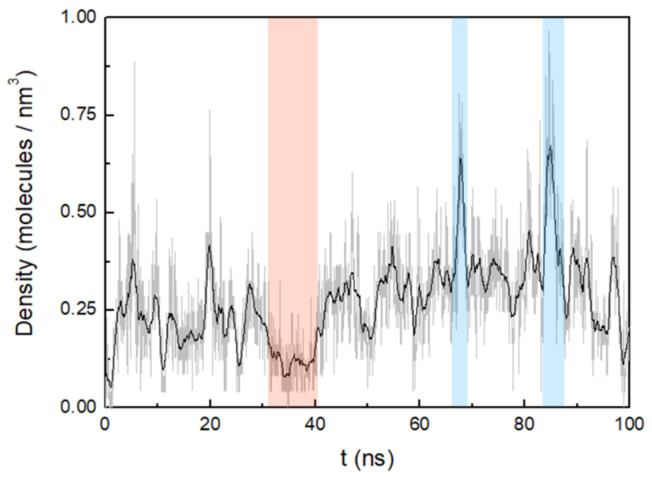
Density of water as a function of time. The most representative pore density maxima and minima have been indicated with blue and red zones, respectively. A running average (window of length 1 ns) was applied.

The water density inside the membrane was measured from the pore centre to 0.3 nm in both directions of the *z*-axis. (Figure 8). Over the period of simulation, three distinct events regarding the water content can be observed in the pore formed by the six octapeptides. The first one occurs up to approximately 34.2 ns, where the average water density is relatively low (0.2 molecules/nm^3^). In the second event, there is a progressive increase in pore water density up to 85 ns, with an average density of 0.3 molecules/nm^3^ (two maxima are observed at 67.6 nm and 84.9 nm). A third event shows a slight decrease in density down to 0.2 molecules/nm^3^ near the end of the simulation. Thus, water molecules seem to penetrate the membrane continuously, but with significant fluctuations.

## 4. Discussion

In order to understand the obvious advantage of the octapeptide KSPFPFAA in an experimental selection process [22], a study of its conformational behaviour in the membrane was carried out. Initially, the peptide conformation in a biphasic system that mimics the octadecylamine–octadecanoic acid bilayer was determined. The octane/water solvent biphasic system has been used as a model as it has been shown to significantly accelerate the conformational relaxation of the peptide as compared to a lipid bilayer [35,36,37,38]. The results obtained in RMSF and RMSD suggest that, in contrast to the peptide–water system, the peptide in a hydrated octane slab exhibits high stability and low flexibility throughout the simulation. It shows a distinctly extended conformation along the bilayer normal and remains largely unfolded. In addition, it was determined that the hydrogen bonds formed by the two end group amino acids (K1 and A8) with the water molecules are principally responsible for keeping the peptide in the unfolded conformation.

In the aggregation study, the peptides in an antiparallel arrangement remained embedded in the membrane throughout the process. In contrast, the parallel arrangement is largely unstable; here, the peptides quickly began to emerge to the membrane surface as larger clusters formed. Obviously, the antiparallel peptide arrangement leads to a significant stabilization via hydrogen bond interactions between the terminal sequences. Particularly, the intermolecular interaction between the amine hydrogen of K1 and the carbonyl oxygen of A8 gives rise to strong and stable trans-membrane aggregates. In addition, the polar head groups of octadecylamine and octadecanoic acid molecules tend to form hydrogen bonds with the A8 residue, which also contribute to the stabilization of the aggregates. Overall, it may also explain for the thermal stabilization of the overall membrane structure which has been observed experimentally by pulsed-field-gradient NMR [22].

In the same experiments, a distinct increase of the membrane permeability for water was observed. This phenomenon is reproduced by the given results from the molecular dynamics calculations. During the simulated aggregation process, it was possible to follow the peptide-induced penetration of water molecules into the membrane. Obviously, it is caused by the formation of central pores within peptide clusters in the membrane. In addition, the influence of residue side chain orientation on pore formation was analysed. It was observed that the charged lysine side chain is stretched, interacting with the membrane head groups and establishing contact with the outside of the membrane [39]. In this way, the lysine side chain enhances the introduction of water molecules into the pore. The hydroxyl side chain of the serine residue is oriented toward the inner side of the pore, allowing water molecules to penetrate and flow into the pore region inside the peptide cluster. The opposite tendency is observed for nonpolar residues, so that the cyclic side chain of proline and the phenyl group of the phenylalanine are oriented toward the hydrophobic tails of octadecylamine and octadecanoic acid. During the time window of the simulation, the water content inside the pore fluctuates dynamically. This observation may be linked to the actual water permeation process which was induced by the peptide in a corresponding experiment [22]. Here, pulsed-field-gradient NMR reveal an increased degree of water permeation through the vesicle membranes with relatively low permeability when the octapeptide is added.

As the most important target for our present experimental studies, we are looking for additional peptides which are developed during long term evolution processes. From these, we expect additional functions which are linked to the survival of the corresponding vesicles. All in all, the simulation results clearly demonstrate why the octapeptide KSPFPFAA has a clear advantage against other peptide structures and therefore is accumulated in the experimental selection process. It forms a well-defined integration product with the bilayer membrane which is stabilizing the resulting composite structure thermodynamically. It enhances the membrane permeability for water, hereby relaxing the destructive osmotic pressure and leading to an increased survival rate of the corresponding vesicles. In order to further verify the results obtained in this study by the formation of peptide aggregates and pores, one could use other experimental techniques such as X-ray diffraction or atomic force microscopy, which provide additional information about the structural effects caused in the membrane. The most exciting result is the fact that relatively complex structures, such as shown in Figure 3 and Figure 7, are formed spontaneously from quite simple educts such as amino acids and basic amphiphiles.

## Figures and Tables

**Table 1:** Percentage of the most characteristic intra-peptide and amino acid residues–water hydrogen bond interactions in the MD trajectory.

**Peptide H-Bonds (hydrated octane slab)/%**							
		**S2-P3**	**F4-F6**	**F6-F8**			
		87	4	5			
**Peptide H-Bonds (water)/%**							
	**K1-S2**	**K1-A8**	**S2-F4**	**S2-A8**	**F4-F6**	**F4-A7**	
	3	23	23	31	6	4	
**Peptide–Water H-Bonds (hydrated octane slab)/%**							
	**K1**	**S2**	**F4**	**A7**	**A8**		
	32	14	3	5	42		
**Peptide–Water H-Bonds (water)/%**							
**K1**	**S2**	**P3**	**F4**	**P5**	**F6**	**A7**	**A8**
26	13	6	5	6	7	9	28

**Table 2:** Percentage of the number of contacts between octadecylamine and octadecanoic acid (head group or tail region) and amino acid residues over the course of the MD trajectory.

**Number of Contacts–Octadecylamine (head)/%**							
**K1**	**S2**	**P3**	**F4**	**P5**	**F6**	**A7**	**A8**
35	9	9	5	5	9	14	14
**Number of Contacts–Octadecanoic Acid (head)/%**							
**K1**	**S2**	**P3**	**F4**	**P5**	**F6**	**A7**	**A8**
16	10	14	16	6	17	7	13
**Number of Contacts–Octadecylamine (tail)/%**							
**K1**	**S2**	**P3**	**F4**	**P5**	**F6**	**A7**	**A8**
12	8	12	19	13	18	8	11
**Number of Contacts–Octadecanoic Acid (tail)/%**							
**K1**	**S2**	**P3**	**F4**	**P5**	**F6**	**A7**	**A8**
8	7	13	21	15	20	8	9

## Data Availability

All data generated during this study are available from the corresponding author upon request.
